# Myocardial Ischemia-Reperfusion Injury: Molecular Mechanisms and Innovative Therapeutic Strategies

**DOI:** 10.31083/RCM51415

**Published:** 2026-09-18

**Authors:** Dong Zhang, Xiaoting Yang, Shengyu Gong, Gang Zhou, Yunyun Zhao, Xiaolin Zhang, Hui Wu

**Affiliations:** ^1^Interventional Diagnosis and Treatment Center, Yichang Central People’s Hospital, 443000 Yichang, Hubei, China; ^2^Institute of Cardiovascular Disease, China Three Gorges University, 443005 Yichang, Hubei, China; ^3^Department of Cardiology, Yichang Central People’s Hospital, 443000 Yichang, Hubei, China; ^4^Central Laboratory, The First College of Clinical Medical Science, China Three Gorges University, 443000 Yichang, Hubei, China; ^5^Pharmaceutical Department, Yichang Central People’s Hospital, 443000 Yichang, Hubei, China

**Keywords:** myocardial ischemia-reperfusion injury, regulated cell death, ferroptosis, pyroptosis, mitochondrial dysfunction

## Abstract

Myocardial ischemia–reperfusion injury (MIRI) is a critical pathological paradox following revascularization in acute myocardial infarction, yet effective clinical interventions remain limited. MIRI involves a complex, interlinked hierarchy of programmed cell death modalities, including ferroptosis, pyroptosis, and cuproptosis, which form a network mediated by the stimulator of interferon genes–glutathione peroxidase 4 (STING–GPX4), NOD-like receptor pyrin domain containing 3–gasdermin D (NLRP3–GSDMD), and sirtuin 3–ATPase copper transporting alpha (SIRT3–ATP7A) axes. The dynamic evolution of the immune microenvironment, mitochondrial dysfunction, and non-coding RNA regulatory networks further compound this. Emerging strategies, including bionanotechnology-enabled targeted delivery, intelligent responsive biomaterials, drug repurposing, genome editing, and precise ion channel modulation, demonstrate significant therapeutic potential. Despite bottlenecks in clinical translation related to delivery efficiency, patient heterogeneity, and efficacy evaluation, multidisciplinary integration and technological innovations are propelling a paradigm shift in MIRI treatment from revascularization alone to comprehensive myocardial protection.

## 1. Introduction

Myocardial ischemia-reperfusion injury (MIRI) is a pathophysiological paradox encountered in patients with acute myocardial infarction undergoing revascularization therapies such as percutaneous coronary intervention (PCI) or coronary artery bypass grafting (CABG) [1]. The paradox lies in the induction of secondary myocardial damage despite timely restoration of blood flow to the occluded coronary artery, leading to serious sequelae including malignant arrhythmias, myocardial stunning, and even heart failure [2]. As a major challenge in contemporary cardiology, the pathological mechanisms underlying MIRI involve a highly intricate network encompassing explosive oxidative stress, calcium ion overload, mitochondrial dysfunction, dysregulated inflammatory cascades, and the activation of programmed cell death pathways such as apoptosis and pyroptosis [3].

The Global Burden of Disease (GBD) 2021 study identifies ischemic heart disease as the leading cause of global mortality [4]. Particularly concerning is the substantial morbidity and mortality associated with its progression to end-stage heart failure, which presents a formidable challenge to public health systems worldwide [5]. Clinical investigations further reveal that approximately 12% of patients sustaining an acute myocardial infarction progress to chronic heart failure within one year, with MIRI serving as a key pathological driver of this adverse outcome [6]. Paradoxically, while contemporary revascularization techniques have markedly improved coronary blood flow restoration, the injury caused by the reperfusion process itself remains a principal constraint on long-term patient prognosis [7]. At present, direct and effective clinical interventions targeting MIRI remain severely limited. Strategies such as ischemic preconditioning or therapeutic hypothermia face significant constraints due to either cumbersome implementation protocols or substantial translational barriers [8]. Consequently, elucidating the molecular mechanisms underlying MIRI pathogenesis and developing effective, precision-targeted therapeutic approaches constitutes an urgent priority in cardiovascular research.

At the molecular level, MIRI is initiated by intracellular acidosis and ionic imbalances during ischemia. Activation of the Na^+^/H^+^ exchanger to correct pH leads to Na^+^ overload, which subsequently reverses the Na^+^/Ca^2+^ exchanger, causing Ca^2+^ overload. Upon reperfusion, the rapid restoration of physiological pH, termed the pH paradox, further exacerbates Ca^2+^ accumulation and triggers opening of the mitochondrial permeability transition pore (mPTP), culminating in cardiomyocyte death. Concurrently, a burst of reactive oxygen species from mitochondrial complexes and nicotinamide adenine dinucleotide phosphate (NADPH) oxidases amplifies oxidative damage to lipids, proteins, and DNA [9]. In addition, reperfusion inflicts direct damage to the coronary microvasculature, leading to microvascular obstruction and intramyocardial hemorrhage. These vascular injuries not only extend the infarct zone but also sustain local inflammation and iron-mediated oxidative stress, further aggravating tissue injury beyond the initial ischemic insult [10].

This review synthesizes current advances at the forefront of MIRI research. It systematically delineates emergent mechanistic insights into novel cell death modalities (ferroptosis, pyroptosis, cuproptosis), immune microenvironment reprogramming, mitochondrial signaling cascades, and the regulatory networks orchestrated by non-coding RNAs. Furthermore, it assesses the translational potential of innovative therapeutic strategies, including bionanotechnology-enabled targeting, stimuli-responsive hydrogel delivery systems, and CRISPR-mediated precision editing. By integrating these multifaceted research trajectories, this work aims to provide investigators with a comprehensive perspective, thereby facilitating the transition from fundamental discoveries to clinical applications.

## 2. Emergent Mechanisms: Novel Perspectives on Molecular Interactions and Cellular Processes

### 2.1 The Multifaceted Networks of Programmed Cell Death

Conventionally conceptualized as being centered on cardiomyocyte apoptosis, MIRI is now recognized through recent research to involve the concerted activation of multiple regulated cell death (RCD) modalities, including ferroptosis, necroptosis, and pyroptosis, which are interwoven into a sophisticated cell death network [11]. This framework encompasses not only the parallel or sequential engagement of diverse RCD pathways but also dynamic intermodal crosstalk that collectively determines cardiomyocyte fate [11]. Ferroptosis: Research has revealed the pivotal role of the stimulator of interferon genes (STING) protein in MIRI. Mitochondrial DNA accumulation during ischemia activates the cyclic GMP-AMP synthase (cGAS)-cyclic GMP-AMP (cGAMP)-STING axis, which prompts STING to bind and degrade glutathione peroxidase 4 (GPX4). This process induces lethal lipid peroxidation independently of the canonical TANK-binding kinase 1 (TBK1)–interferon regulatory factor 3 (IRF3) pathway, unveiling a novel therapeutic target [12]. Inflammatory pyroptosis: NOD-like receptor pyrin domain containing 3 (NLRP3) inflammasome activation constitutes a central event wherein reperfusion triggers NLRP3-mediated caspase-1 recruitment, proteolytic cleavage of gasdermin D (GSDMD), and consequent pore-driven lytic demise with proinflammatory cytokine (IL-1β/IL-18) release [13,14]. Ginsenoside Rg1 mitigates myocardial damage by suppressing the NLRP3/caspase-1/GSDMD axis, while S100A9 gene exacerbates pyroptosis via NLRP3 upregulation [15]. Cuproptosis: Hemodynamic stress induces copper ion dyshomeostasis, provoking novel copper-dependent death [16]. Research indicates that the RIPK1/RIPK3/MLKL pathway can be modulated. Targeted inhibition of necroptosis protects against myocardial ischemia/reperfusion injury [17]. These findings demonstrate that ferroptosis, pyroptosis, and cuproptosis form an interlinked hierarchical network in MIRI, with the STING-GPX4, NLRP3-GSDMD, and RIPK1/RIPK3/MLKL axes constituting a tripartite system that redefines the therapeutic landscape (Table 1, Ref. [12,13,14,16,17]).

**Table 1.** **Cell death modalities in myocardial ischemia-reperfusion injury**.

Mode of cell death	Key triggering factors	Characteristic biomarkers	Intervention targets
Ferroptosis [12]	Lipid peroxidation accumulation, GPX4 inactivation	Decreased GSH/GSSG ratio, increased ACSL4	STING inhibitors, iron chelators
Pyroptosis [13,14]	Activation of NLRP3 inflammasome	Activation of Caspase-1, cleavage of GSDMD	NLRP3 inhibitors, S100A9 antagonists
Cuproptosis [16]	Overload of mitochondrial Cu^2+^	Aggregation of FDX1, abnormal lipidated proteins	SIRT3 activators, copper chelators
Necroptosis [17]	Activation of RIPK1/RIPK3	MLKL phosphorylation, membrane integrity disruption	Necrostatin-1, RIPK3 inhibitors

GPX4, glutathione peroxidase 4; NLRP3, NOD-like receptor pyrin domain containing 3; RIPK1, receptor-interacting protein kinase 1; GSH, glutathione; GSSG, Oxidized Glutathione; ACSL4, acyl-CoA synthetase long-chain family member 4; GSDMD, gasdermin D; FDX1, ferredoxin 1; MLKL, mixed lineage kinase domain-like protein; STING, stimulator of interferon genes.

### 2.2 Dynamic Remodeling of the Immune-Inflammatory Microenvironment

The immune-inflammatory microenvironment during MIRI undergoes precisely orchestrated dynamic evolution. This temporal evolution can be categorized into three distinct yet overlapping phases. The hyperacute phase (0–6 hours post-reperfusion) is characterized by explosive neutrophil infiltration, formation of neutrophil extracellular traps (NETosis), and massive release of damage-associated molecular patterns (DAMPs) including high mobility group box 1 (HMGB1), adenosine triphosphate (ATP), and mitochondrial DNA, which activate innate immunity through toll-like receptor 4 (TLR4) and NLRP3 pattern recognition receptors. The acute phase (6–72 hours) witnesses dominant infiltration of Ly6C++ inflammatory monocyte-derived macrophages, NLRP3 inflammasome activation with caspase-1-dependent pyroptosis, and prominent M1 macrophage polarization secreting interleukin-1β (IL-1β) and tumor necrosis factor alpha (TNF-α). The subacute to chronic phase (72 hours to 7 days) is marked by upregulation of regulatory T cells (Treg) and CD206+ M2 macrophages that secrete IL-10 and TGF-β, mediating inflammatory resolution, fibroblast activation, and tissue repair. Persistent mitochondrial DNA activation of the cGAS-STING pathway during this phase can, however, impede reparative processes through type I interferon responses. During the ischemic phase, hypoxia prompts cardiomyocytes to release DAMPs (e.g., HMGB1, ATP, mitochondrial DNA), which activate innate immunity via pattern recognition receptors including TLR4 and NLRP3 [18,19]. Within hours of reperfusion initiation, neutrophils constitute the primary infiltrating population, exacerbating endothelial damage and promoting microvascular obstruction through reactive oxygen species (ROS) generation, protease release, and NET formation [20]. Concurrently, resident cardiac macrophages polarize toward a proinflammatory M1 phenotype, secreting IL-1β and TNF-α to further recruit monocytes/macrophages [21]. At mid-stage reperfusion, monocyte-derived inflammatory macrophages dominate the landscape, amplifying tissue injury via NLRP3 inflammasome-mediated pyroptosis and consequent IL-18 release [22,23]. Notably, contemporary research reveals an intrinsic homeostatic shift during later stages, characterized by concomitant upregulation of Treg and CD206^+^ M2 macrophages that secrete IL-10 and transforming growth factor beta (TGF-β) to suppress excessive inflammation and facilitate repair [24]. However, persistent mitochondrial DNA (mtDNA) activation of type I interferon responses through the cGAS-STING pathway impedes reparative processes [25]. Emerging therapeutic strategies consequently emphasize chronologically targeted interventions: early-phase inhibition of NETosis (via PAD4 inhibitors) or NLRP3 activation, coupled with late-phase enhancement of Treg functionality or macrophage phenotypic switching toward reparative states to reestablish immune homeostasis [26,27]. Collectively, the MIRI immune microenvironment manifests a triphasic trajectory—proinflammatory burst, sustained injury, and delayed resolution—rendering temporally precise immunomodulation a frontier in therapeutic innovation.

NLRP3 inflammasome activation is a key mediator of myocardial ischaemia–reperfusion injury (IRI) and endothelial dysfunction. Upon IRI, damage signals trigger NLRP3 assembly, leading to caspase-1 activation, gasdermin D-mediated pyroptosis, and release of IL-1β and IL-18, which exacerbate cardiomyocyte death and vascular inflammation [28]. Endothelial cells are particularly vulnerable: NLRP3 activation promotes adhesion molecule expression, leukocyte infiltration, and reactive oxygen species production, while reducing nitric oxide bioavailability, culminating in coronary microvascular dysfunction and the no-reflow phenomenon [29]. Furthermore, targeting pyroptosis with NLRP3 inhibitors reduces endothelial cell death and inflammation, thereby preserving cardiac function after IRI [30]. These findings support NLRP3 inhibition as a promising strategy against myocardial IRI and associated endothelial dysfunction, though further clinical validation is required.

### 2.3 Mitochondrial Dysfunction and Disruption of Ionic Homeostasis

The ischemic phase initiates a pathophysiological cascade wherein ATP depletion inactivates the Na^+^/K^+^-ATPase, precipitating intracellular Na^+^ accumulation that propels Ca^2+^ influx via reverse-mode Na^+^/Ca^2+^ exchanger (NCX) operation, thereby instigating cytosolic calcium overload [31,32]. Upon reperfusion, hyperactivation of the mitochondrial calcium uniporter (MCU) induces profound mitochondrial Ca^2+^ sequestration, triggering irreversible opening of the mPTP, an event that collapses mitochondrial membrane potential, terminates ATP synthesis, and unleashes fulminant reactive oxygen species generation [33]. These processes establish a self-amplifying circuit. Mitochondrial ROS inhibit sarco/endoplasmic reticulum Ca^2+^-ATPase (SERCA) activity through oxidative modification of critical cysteine residues (Cys674 and Cys675), thereby impairing calcium reuptake into the sarcoplasmic reticulum and exacerbating cytosolic calcium overload. Conversely, accumulated cytosolic Ca^2+^ forms complexes with calmodulin (Ca^2+^-CaM), which directly binds and activates the p47phox regulatory subunit of NADPH oxidase 2 (NOX2), potentiates NADPH oxidase-dependent ROS generation, and thereby establishes a self-perpetuating ROS-Ca^2+^ vicious cycle that progressively amplifies myocardial injury. Additionally, aberrant dynamin-related protein 1 (Drp1) phosphorylation at Ser616 by Ca^2^⁺/calmodulin-dependent protein kinase II (CaMKII) and protein kinase C (PKC) drives pathological mitochondrial fission, whereas peroxisome proliferator-activated receptor gamma coactivator 1-alpha (PGC-1α)-dependent phosphorylation at Ser65 regulates the fusion-fission balance. Mitochondrial ROS inhibit sarco/endoplasmic reticulum Ca^2+^-ATPase activity, exacerbating calcium leakage, while cytosolic Ca^2+^ activates NOX2 oxidase to potentiate oxidative stress [34]. Concurrently, impaired PTEN-induced kinase 1 (PINK1)-mediated phosphorylation of ubiquitin (Ub-Ser65) disrupts Parkin recruitment, culminating in defective mitophagy and accumulation of fragmented mitochondria [35]. Mitochondrion-specific ferroptosis amplifies injury through synergistic membrane destabilization: acyl-CoA synthetase long-chain family member 4 (ACSL4)-catalyzed peroxidation of polyunsaturated fatty acid phospholipids converges with free iron-driven Fenton chemistry to compromise organellar integrity [36]. Current therapeutic strategies target this multi-nodal axis: MCU complex modulation (e.g., MCUi-11) attenuates calcium overload [37]; Transcription factor EB (TFEB) activation (e.g., Urolithin A) enhances mitophagic flux [38,39]; and iron chelation (e.g., deferiprone, DFP) counters ferroptotic cascades [40]. The mitochondrial-ion nexus in MIRI thus manifests as an escalating pathological network of calcium overload, mPTP opening, ROS burst, dynamics imbalance, and ferroptosis, wherein chronologically coordinated multi-target intervention constitutes the cornerstone of translational endeavors.

### 2.4 Fine-Tuned Regulatory Circuitry of Non-Coding RNAs

Building upon the mechanistic foundations established in the preceding sections, non-coding RNA (ncRNA) networks constitute a critical regulatory layer that modulates the pathological processes described above. As previously described, mitochondrial ROS generation and calcium dysregulation constitute central drivers of MIRI; conversely, as elaborated in Section 2.1, ferroptotic and pyroptotic cascades execute cardiomyocyte demise. The ncRNA systems described herein do not operate in isolation but function as dynamic modulators of these established pathways. For instance, lncRNA H19-driven enhancer of zeste homolog 2 (EZH2)-mediated epigenetic silencing of superoxide dismutase 2 (SOD2) directly amplifies the mitochondrial ROS burst cascade, while circFASN-mediated NF-kappaB activation propagates the inflammatory signaling that drives NLRP3-GSDMD pyroptosis. Thus, ncRNAs provide fine-tuned, spatiotemporal regulatory control over the MIRI pathophysiological network. Non-coding RNAs orchestrate spatiotemporally defined regulatory networks that dynamically modulate both pathological and reparative pathways during MIRI [41,42]. During the initial ischemic phase, rapid upregulation of the long non-coding *RNA H19 *functions as a molecular sponge, sequestering *miR-877-3p* and thereby derepressing its target EZH2—a histone methyltransferase that epigenetically silences the antioxidant gene SOD2, exacerbating oxidative stress [43,44]. Upon reperfusion, circular RNA *CircFASN* engages in competitive endogenous RNA (*ceRNA*) crosstalk by binding *miR-652-3p*, which liberates TNF receptor-associated factor 3 (TRAF3) to activate the nuclear factor kappa-B (NF-*κ*B) pathway and propagate inflammatory cascades [45]. Concurrently, *miR-34a-5p* represses mitochondrial biogenesis by targeting SIRT1, consequently disrupting *PGC-1α*-mediated energy metabolism [46,47]. A breakthrough study reveals that exosomally shuttled *lncRNA MALAT1* potentiates macrophage M1 polarization through HuR protein-mediated stabilization of TNF-α mRNA, amplifying myocardial damage [48]. Conversely, during the reparative phase, *CircHIPK3 *promotes angiogenesis by adsorbing *miR-330-5p* to activate the *VEGFA/PDGFRβ* axis, while *miR-214-3p* attenuates calcium overload through *CaMKIIδ* suppression [49,50]. Notably, *lncRNA KCNQ1OT1* coordinates antioxidant defenses by assembling ribonucleoprotein complexes with fused in sarcoma (FUS) protein to enhance nuclear factor erythroid 2-related factor 2 (Nrf2) transcriptional activity [51]. Emerging therapeutic paradigms exploit engineered exosomes for targeted delivery of protective circRNAs (e.g., *CircSnx12*-mediated NLRP3 inflammasome inhibition) or locked nucleic acid (LNA)-based silencing of pro-apoptotic miRNAs (e.g., *miR-15b* targeting BCL2), demonstrating unprecedented precision in RNA modulation [52,53]. Collectively, non-coding RNAs govern MIRI progression through multilayered mechanisms—*ceRNA* networks, epigenetic remodeling, and ribonucleoprotein assembly—establishing spatiotemporally resolved interventions as the vanguard of translational cardiology. Furthermore, the ncRNA regulatory architecture forms an indispensable integrative component of the MIRI pathophysiological framework described throughout this review. The ceRNA networks (e.g., H19/miR-877-3p/EZH2, circFASN/miR-652-3p/TRAF3) directly modulate the STING-GPX4 ferroptotic axis and NLRP3-GSDMD pyroptotic cascade. Concurrently, epigenetic remodeling through lncRNA-protein assemblies (e.g., KCNQ1OT1-FUS-Nrf2 complexes) governs the transition between the proinflammatory burst phase (Phase I) and reparative resolution phase (Phase III) within the immune microenvironment described. By interfacing with mitochondrial signaling, cell death execution, and immune modulation, ncRNAs establish a multilayered regulatory nexus that coordinates the spatiotemporal progression of MIRI.

Cardioprotective strategies targeting mitochondrial dynamics have shown efficacy in preclinical models but face translational challenges. Hernandez-Resendiz et al. [54] detail how MIRI disrupts mitochondrial fission/fusion balance, and interventions such as ischemic preconditioning (IPC), postconditioning (IPost), and remote ischemic conditioning (RIC) preserve mitochondrial morphology, reducing infarct size. However, Kleinbongard et al. [55] demonstrate substantial differences between animal and human settings. In pigs, IPC consistently reduces infarct size under controlled conditions with healthy, young animals and standardized protocols, whereas clinical application is hindered by patient comorbidities, concomitant medications, and logistical constraints. For IPost, precise algorithm application at reperfusion onset is feasible in experimental models but difficult to achieve in emergency percutaneous coronary intervention. RIC shows robust cardioprotection in healthy pigs, yet large clinical trials have yielded neutral results. Thus, the therapeutic potential of vesicles and gases requires further validation.

In summary, MIRI involves a sophisticated network of regulated cell deaths (ferroptosis, pyroptosis, cuproptosis) mediated by STING-GPX4, NLRP3-GSDMD, and SIRT3-ATP7A axes. The immune response follows a triphasic trajectory: proinflammatory burst, sustained injury, and delayed resolution. Mitochondrial dysfunction manifests as calcium overload leading to mPTP opening, ROS burst, and ferroptosis. Non-coding RNAs orchestrate spatiotemporal regulation through ceRNA networks and epigenetic remodeling (Fig. 1). These findings collectively demonstrate that chronologically coordinated multi-target interventions targeting these interconnected pathways represent the frontier of therapeutic innovation.

**Fig. 1.**
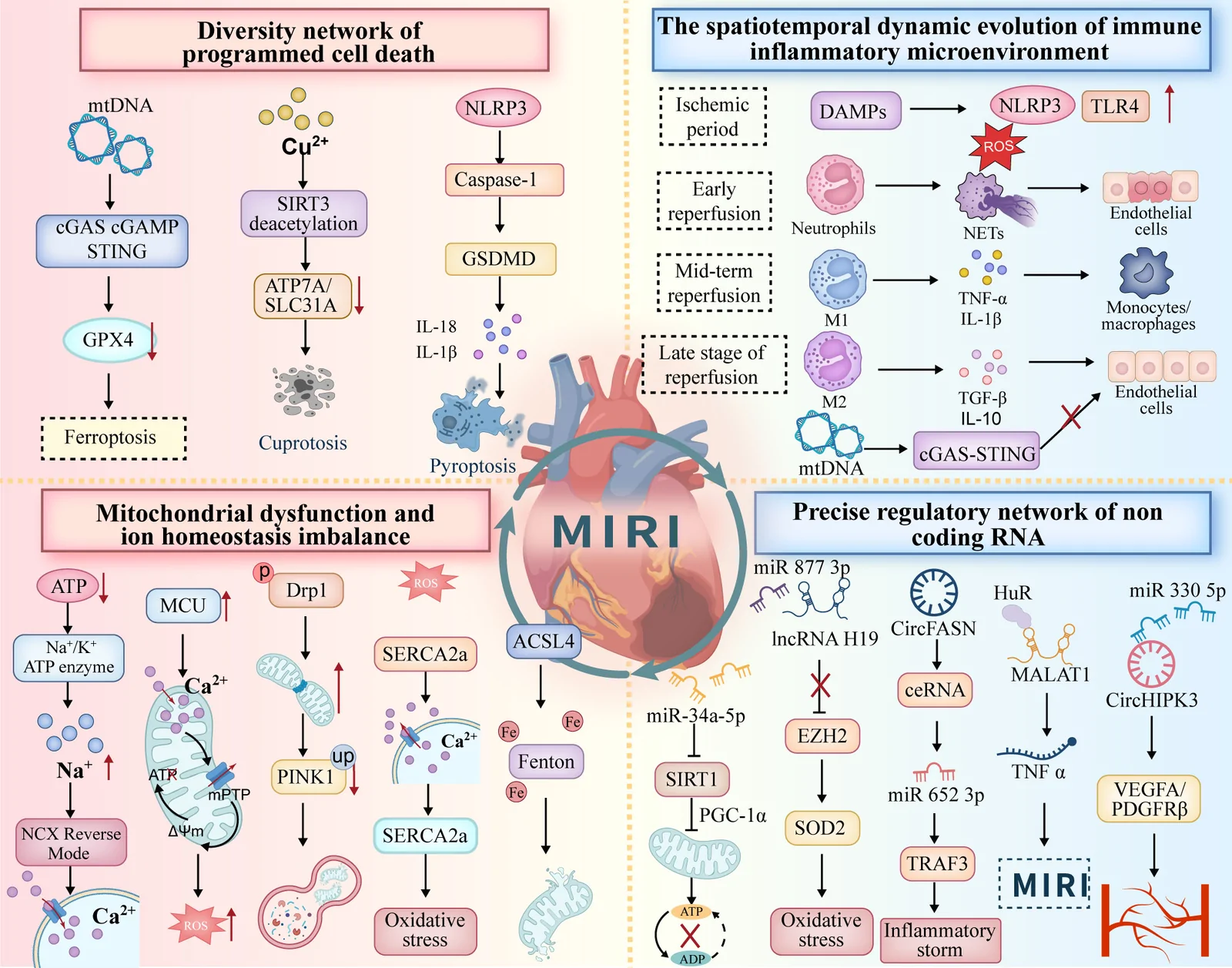
**Temporal and spatial dynamic evolution of immune inflammatory microenvironment and programmed cell death regulatory network in myocardial ischemia-reperfusion injury**. MIRI, myocardial ischemia-reperfusion injury; DAMPs, damage-associated molecular patterns; cGAS-STING, cyclic GMP-AMP synthase-stimulator of interferon genes; SOD2, superoxide dismutase 2; MCU, mitochondrial calcium uniporter; TRAF3, TNF receptor-associated factor 3. ↑, increase in expression level; ↓, decrease in expression level; ×, no direct regulatory effect. This image was created using Adobe Acrobat DC software.

## 3. Therapeutic Frontiers: Innovative Paradigms From Targeted Delivery to Immunomodulation

### 3.1 Precision-Guided Delivery Systems Enabled by Bionanotechnology

Translating mechanistic insights, specifically the identification of the STING-GPX4 ferroptotic axis and NLRP3-GSDMD pyroptotic cascade as critical drivers of cardiomyocyte death, into clinical applications necessitates precision delivery systems capable of specifically targeting these molecular nodes within the ischemic myocardium. The pursuit of precision therapeutics for MIRI requires overcoming formidable physiological barriers and spatiotemporal constraints, wherein bionanotechnology has forged innovative avenues through active targeting, stimulus-responsive drug release, and synergistic immunomodulation [56]. Cell-membrane camouflage represents a significant advance: platelet-membrane-coated nanoparticles (PNPs) leverage retained CD47 signaling and P-selectin-mediated vascular tropism to prolong circulatory persistence and achieve ischemic myocardium enrichment (preclinical evidence) [57]. Engineered exosomes demonstrate distinctive advantages: vascular cell adhesion molecule-1 (VCAM-1)-targeted peptides functionalized onto macrophage-derived vesicles enable efficient NLRP3 inflammasome silencing via siRNA delivery while harnessing inherent immunoregulatory properties to remodel the inflammatory microenvironment [58,59]. Stimuli-responsive vectors achieve spatiotemporal control: ROS-labile hydrogels encapsulating mesoporous silica nanoparticles orchestrate burst release of mitochondrial-protective peptide SS-31 upon encountering early reperfusion oxidative stress, concurrently enabling sustained liberation of pro-angiogenic miR-24-3p [60,61]. Innovative cascade functionalization is exemplified by neutrophil-mimetic nanovehicles co-loaded with CaMKIIδ inhibitor KN-93, which exploit CD11b/CD18 integrin-mediated inflammatory endothelium targeting followed by membrane fusion-mediated tissue penetration for cardiomyocyte-specific calcium overload blockade [62,63]. Mitochondrial-targeted delivery constitutes a pivotal advancement: triphenylphosphonium (TPP^+^)-conjugated metal-organic framework (MOF) nanocarriers utilize charge-driven mitochondrial matrix accumulation to precisely deploy MitoQ, quenching mitochondrial ROS and restoring electron transport chain integrity (preclinical, porcine model) [64,65]. Current frontiers explore combinatorial gene delivery: lipid-polymer hybrid nanoparticles co-encapsulating circRNA (CircSnx12) and mRNA (VEGF) exploit pH-responsive charge-reversal layers for infarction zone-specific release, concurrently suppressing pyroptosis and promoting vasculogenesis [66]. These biomimetic platforms, transcending biological barriers through molecular disguise, achieving lesion-precise drug liberation via microenvironmental responsiveness, and enhancing therapeutic efficacy via organellar targeting, are catalyzing a shift toward multiscale precision intervention spanning cellular, molecular, and suborganellar dimensions in MIRI management.

Several critical limitations of bionanotechnology-enabled delivery must be acknowledged for realistic translational assessment. First, nanocarriers undergo hepatic Kupffer cell and splenic macrophage sequestration, thereby facing a major challenge following systemic injection [67]. In addition, the immunogenicity of repeated exosome administration remains incompletely characterized, with potential risks including anti-PEG antibody formation and immune responses to membrane-associated proteins [68]. Lastly, the regulatory complexity of classifying engineered exosomes as biological products versus drug delivery vehicles presents unresolved issues [69].

### 3.2 Spatiotemporally Programmed Therapeutic Biomaterials

The intelligent biomaterials described in this section are engineered to sense and respond to the specific pathological cues—namely, the ROS-enriched inflammatory microenvironment of early reperfusion and the calcium-overloaded mitochondrial dysfunction that characterizes progressive myocardial injury. The therapeutic paradigm for MIRI is evolving from passive intervention toward active regeneration, with intelligent biomaterials emerging as frontline modalities capable of autonomously sensing pathological cues and executing site-specific reparative functions [70,71]. ROS-responsive hydrogels exemplify this approach: they undergo rapid dissolution in the oxidative microenvironment of early reperfusion, enabling precision release of encapsulated mitochondrial-protective peptide SS-31 alongside sustained liberation of pro-angiogenic miR-21, thereby achieving temporally prioritized cytoprotection followed by regenerative processes [72,73]. Self-assembling nanofibers demonstrate distinctive capabilities: peptide-based matrices (e.g., RADA16) injected into myocardium spontaneously organize into three-dimensional nanoscale networks under acidic ischemic conditions, functioning as tissue-adhesive nanoscaffolds that concurrently deliver stromal-derived factor-1α (SDF-1α) to orchestrate stem cell homing [74,75]. More sophisticated designs include dual-signal responsive microneedle patches: pH-sensitive polymeric bases adhere to infarcted tissue in acidic microenvironments, while matrix metalloproteinase-2 (MMP-2)-cleavable tips deliver anti-IL-10 antibodies for immunomodulation alongside conductive gold nanowires that restore electrophysiological synchrony [76,77]. To counteract lethal calcium overload, engineered hydrogels incorporating calcium-buffering proteins (e.g., calbindin-D28k) function as molecular sponges, sequestering cytosolic Ca^2+^ and preventing mitochondrial permeability transition pore oscillation [78]. Current frontiers focus on *in situ* therapeutic factories: metal-organic frameworks (MOFs) loaded with photosensitizer chlorin e6 (Ce6) generate localized ROS under near-infrared irradiation, triggering self-disassembly and controlled release of hydrogen sulfide (H_2_S) to simultaneously induce vasodilation and suppress apoptosis [79]. Meanwhile, conductive polymer scaffolds (e.g., polypyrrole/chitosan) not only transmit electrical impulses to stabilize cardiac rhythm but also promote neovascularization through porous architectures, achieving tripartite repair through electrical stabilization, structural reinforcement, and regenerative potentiation [80,81]. These endogenous bioresponsive systems function as implantable microphysicians: sensing injury signatures (pH/enzymes/ROS), deploying targeted therapeutics, and reconstructing tissue integrity, establishing a dynamic shield for cardiac protection.

### 3.3 Polypharmacological Drug Repurposing and Bioactive Phytochemicals

The polypharmacological agents highlighted in this section are selected for their demonstrated capacity to concurrently modulate multiple pathological cascades spanning ferroptosis (via ACSL4/GPX4), pyroptosis (via NLRP3 inflammasome), and mitochondrial dysfunction (via SIRT3/PGC-1α pathways), thereby achieving integrated cardioprotection through multi-target engagement. The pathophysiological complexity of MIRI necessitates concurrent modulation of multiple pathological cascades, wherein polypharmacological drug repurposing and bioactive phytochemicals emerge as strategically efficient modalities by virtue of their inherent multi-target engagement [82,83]. The antidiabetic sodium-glucose cotransporter 2 (SGLT2) inhibitor empagliflozin exemplifies this paradigm, reducing infarct size by 30% through dual AMP-activated protein kinase (AMPK) activation and sodium-hydrogen exchanger isoform 1 (NHE1) inhibition, simultaneously mitigating sodium-calcium overload and augmenting mitochondrial autophagy (Phase III clinically validated) [84,85,86]. The antimalarial chloroquine has been repurposed as a context-dependent autophagy modulator: low doses enhance clearance of damaged mitochondria via TFEB activation, whereas higher concentrations suppress excessive autophagic flux, albeit with requisite vigilance toward proarrhythmic potential [87,88]. Among natural compounds, Shikonin demonstrate dual efficacy by targeting the ACSL4/GPX4 axis to suppress ferroptosis while concurrently inhibiting NLRP3 inflammasome assembly, thereby integrating anti-inflammatory and antioxidant functions [89,90]. Tanshinone I (derived from Salvia miltiorrhiza) sustains mitochondrial metabolism through SIRT3 activation and potentiates ROS clearance via Nrf2 pathway enhancement, with its nanocrystalline formulation achieving a fivefold increase in oral bioavailability [91,92]. Garlic has been shown to exert substantial medicinal effects and is considered to be one of the best disease-preventative foods [93]. Emerging evidence has shown that hydrogen sulfide (H_2_S) has cardioprotective and cytoprotective properties. The active metabolite in garlic, allicin, is readily degraded into organic diallyl polysulfides that are potent H_2_S donors in the presence of thiols. Myocardial-targeted delivery enables site-specific release of H_2_S, eliciting microvascular dilation and inhibition of mitochondrial permeability transition pore opening [94]. Andrographolide attenuates calcium overload through allosteric CaMKIIδ binding while suppressing neutrophil extracellular trap formation, with its liposomal formulation advancing to Phase I clinical trials [95]. These multimodal therapeutic agents function as pharmacological multitools: repurposed antidiabetics confer cardioprotection, repositioned antimalarials fine-tune autophagy, and phytochemicals simultaneously counteract inflammation and preserve mitochondrial integrity, offering a strategic advantage that circumvents the protracted timelines of de novo drug development (Table 2, Ref. [84,85,86,87,88,89,90,91,92,94,95]).

**Table 2.** **Comparative analysis of polypharmacological agents**.

Pharmacological compounds	Category	Core mechanism	Advantages	Limitations
Empagliflozin [84,85,86]	SGLT2 inhibitor	Activation of AMPK↑/Inhibition of NHE1→Reduction of calcium overload	Cardiovascular protection with clinical evidence	May increase the risk of ketoacidosis
Chloroquine [87,88]	Antimalarial drug	Low-dose promotes TFEB autophagy/High-dose inhibits excessive autophagy	Low cost, convenient administration	Narrow therapeutic window, high doses can cause arrhythmias
Shikonin [89,90]	Natural naphthoquinone	Inhibition of ACSL4 ferroptosis + Suppression of NLRP3 inflammasome	Dual pathways, new type of nano-formulation with high efficacy	Poor water solubility, requires carrier delivery
Tanshinone I [91,92]	Danshen extract	Activation of SIRT3 mitochondrial function + Enhancement of Nrf2 antioxidant	Multi-organ protection, high safety	Low oral bioavailability (<10%)
Garlic [94]	Natural polyphenol prodrug	Targeted release of H_2_S→Dilation of blood vessels/Inhibition of mPTP opening	Microenvironment-responsive prodrug, low side effects	Fast metabolism in the body, requires frequent dosing
Andrographolide [95]	Diterpenoid	Inhibition of CaMKIIδ↓/Calcium overload + Blockade of NET formation	Immune-ion double regulation, lipid body prodrug with advantages	High dose hepatotoxicity, limited clinical data

→: indicates causing or promoting; ↑: indicates increase or upregulation; ↓: indicates decrease or inhibition.

### 3.4 Precision Genome Editing and Epigenetic Governance

Genome editing and epigenetic governance strategies offer the potential for permanent correction of pathogenic mechanisms, particularly the CaMKIIδ-driven calcium overload and mitochondrial fission, and for epigenetic remodeling of protective gene expression programs (SIRT3 antioxidant axis, Nrf2 pathway). Therapeutic paradigms for MIRI are evolving from symptomatic management toward foundational correction, with genome editing and epigenetic governance enabling precise interventions through direct pathogenic gene modification or transcriptional reprogramming [96,97,98]. CRISPR/Cas9-mediated knockout represents a significant advance: targeted silencing of the calcium/calmodulin-dependent protein kinase IIδ (CaMKIIδ) gene significantly attenuates calcium overload and mitochondrial fission, achieving 40% infarct reduction in large-animal models (preclinical, large-animal models) [49,89,99]. Safer epigenetic editing obviates DNA sequence alteration: dCas9-TET1 fusion proteins direct hydroxymethylation to the SIRT3 promoter, enhancing mitochondrial antioxidant capacity with effects persisting for at least 3 months (preclinical, rodent models) [100]. For inflammatory cascades, dynamic histone methylation emerges as a novel target: EZH2 methyltransferase inhibition opens chromatin architecture at the SOD2 antioxidant locus, while JMJD3 demethylase inhibitors suppress TNF-α transcription through promoter compaction [101,102,103,104]. Innovative RNA epitranscriptomic editing enables concurrent modulation: FTO demethylase erases m^6^A modifications on pivotal lncRNAs (e.g., MALAT1), disrupting their cytokine-stabilizing function via exosome-enabled cardiac delivery [105,106]. Clinical translation is accelerated by non-viral vectors: ionizable lipid nanoparticles (LNPs) co-delivering SaCas9 mRNA and miR-34a-targeting sgRNA achieve 70% cardiac-specific reduction in apoptotic genes following intravenous administration [107,108,109]. Concurrently, phytochemical-mediated epigenetic remodeling offers translational efficiency: andrographolide inhibits DNMT1 methylation, reactivating silenced Nrf2 pathway genes to potentiate endogenous antioxidant defenses through oral bioavailability [110]. Genome editing provides surgical precision for excising pathogenic elements (e.g., CaMKIIδ), while epigenetic orchestrators fine-tune protective gene expression (SIRT3/Nrf2) via methylation markers (m^6^A/histones), collectively enabling foundational-level genomic rectification for cardiovascular repair. Several safety considerations warrant careful evaluation before clinical translation of genome editing for MIRI. Off-target DNA cleavage remains a principal concern, as even rare off-target indels in long-lived cardiomyocytes could accumulate pathological consequences over time [111]. The permanence of genetic modifications poses unique ethical and reversible clinical challenges distinct from pharmacological or epigenetic interventions, which can be discontinued if adverse effects emerge. Furthermore, both viral vectors and non-viral vectors can elicit innate inflammatory and adaptive immune responses, potentially limiting repeat dosing and complicating safety monitoring. Regulatory frameworks for *in vivo* CRISPR therapeutics remain actively evolving, with the FDA requiring comprehensive genotoxicity studies, extended biodistribution assessments, and long-term follow-up protocols prior to Investigational New Drug (IND) application approval.

### 3.5 Precision-Targeted Therapeutics Modulating Ionic Flux

The precision ion channel therapeutics discussed in this section directly target the ionic homeostasis collapse, specifically interrupting the calcium overload to mPTP opening to ROS burst cascade that constitutes the core pathophysiological driver of reperfusion injury. MIRI fundamentally manifests as a collapse of ionic homeostasis, rendering precision-targeted modulation of specific ion channels a critical frontier for electrophysiological restitution [83,112]. The inciting pathophysiological event is calcium overload during early reperfusion: novel allosteric inhibitors such as GP-5 stabilize the closed conformation of ryanodine receptor 2 (RyR2) channels, thereby preventing sarcoplasmic reticulum calcium leakage, functionally analogous to sealing a dysfunctional valve, while combinatorial administration of the reverse-mode Na^+^/Ca^2+^ exchanger inhibitor ORM-10962 reduces cytosolic calcium concentration by 40% [113]. For mitochondrial calcium dysregulation, the small molecule MCUi-11 selectively blocks the MCU, averting pathological mPTP opening through a mechanism akin to installing a preventive molecular fuse, which significantly attenuates cardiomyocyte apoptosis (preclinical, Phase II candidate) [114]. Dynamic sodium channel regulation represents an emerging focus: enhanced late sodium current (I_NaL) during reperfusion drives pathological sodium accumulation, wherein the targeted inhibitor GS-967, exerting cardiolipin-specific binding, not only suppresses I_NaL but also preserves bioenergetic metabolism by rectifying mitochondrial membrane phospholipid composition [115]. To counteract lethal arrhythmogenesis, the ATP-sensitive potassium channel (mitoK_ATP) agonist nicorandil has been reformulated as nanocrystals, enabling mitochondrial-targeted channel activation to stabilize membrane potential and quench reactive oxygen species generation [116]. Pioneering advances include dual-function channel modulators: compound CXL-1020 activates SUR2A subunits (K_ATP channels) to induce vasodilation while concurrently inhibiting acid-sensing ion channel 1a (ASIC1a) to mitigate reperfusion acidosis [117]. Optogenetic approaches achieve spatiotemporal precision: adenoviral delivery of the light-sensitive calcium-translocating channelrhodopsin (CatCh) permits blue light-triggered augmentation of calcium influx to enhance contractility without systemic pharmacological sequelae [118]. These molecular gatekeepers restore cardiac electrophysiological integrity through channel-specific interventions: sealing calcium leaks (RyR2/NCX), closing aberrant sodium gates (I_NaL), and opening potassium conduits (mitoK_ATP), to reinstate ionic equilibrium at the organ level.

## 4. Unresolved Challenges and Evolving Frontiers

### 4.1 Animal-Human Translational Discrepancy

Despite decades of research, the translation of cardioprotective strategies for myocardial ischemia-reperfusion injury remains an unmet challenge. A key unresolved issue is the discordance between robust preclinical data and neutral clinical outcomes, as highlighted by the CONDI-2/ERIC-PPCI cardiovascular magnetic resonance (CMR) substudy, which found that limb remote ischemic conditioning (RIC) did not reduce infarct size or improve left ventricular ejection fraction in low-risk STEMI patients treated by primary percutaneous coronary intervention [119]. This disparity underscores the need for more rigorous preclinical assessment. The IMPACT criteria advocate for systematic *in vivo* evaluation incorporating comorbid and aged animal models before clinical trials, a step often overlooked [120]. Furthermore, significant variability exists regarding biological sex and ageing. The interaction of cardiovascular risk factors, such as advanced age and diabetes, with MIRI is complex; these factors frequently attenuate or abolish the cardioprotective efficacy of conditioning strategies, yet many preclinical studies fail to account for them [121]. Similarly, most animal studies use young, healthy models, while clinical patients are typically older with multimorbidities. Divergent responses between human and animal models also persist. The RIC-AFRICA trial aims to address this by testing RIC in higher-risk, thrombolysis-treated STEMI patients in sub-Saharan Africa, suggesting that benefit may be reserved for populations with delayed reperfusion and greater baseline risk [122]. Future progress requires harmonizing preclinical and clinical endpoints, including sex-specific analyses, and validating therapies across species and disease contexts.

### 4.2 Translational Bottlenecks and Implementation Barriers

Despite substantial preclinical advances in MIRI therapeutics, clinical translation confronts three fundamental bottlenecks. Foremost is inefficient drug delivery: over 90% of nanocarriers undergo hepatic/splenic sequestration, with ischemic myocardium-targeting efficiency remaining below 5% (mitochondria-directed peptide SS-31 exhibits less than 1% cardiac accumulation in large-animal models), compounded by microvascular destruction that further impedes perfusion [123,124]. Disease heterogeneity presents greater challenges: in diabetic MIRI patients, hyperglycemia-induced O-GlcNAcylation alters CaMKIIδ activity, diminishing calcium overload intervention efficacy by 63 percentage points versus non-diabetic cohorts, while age-related mitophagic defects attenuate responses to Parkin-targeting agents [125,126,127]. Critical disjunction in endpoint metrics underlies translational failures: although infarct size reduction remains the preclinical gold standard. However, that that part of phase III trial failures demonstrated preserved infarct-sparing effects yet failed to improve hard clinical endpoints including cardiovascular mortality and heart failure hospitalization (exemplified by mitoK_ATP opener nicorandil’s inability to reduce long-term mortality) [128]. Moreover, therapeutic window disparities remain gravely overlooked: whereas animal models typically administer interventions immediately upon reperfusion, clinical reperfusion averages a 3.5-hour delay post-symptom onset, permitting irreversible inflammatory cascades that abrogate NLRP3 inhibitor efficacy [129,130,131]. Emerging solutions show promise: patient-derived cardiac organoids (iPSC-CMs) successfully model individualized pathophysiology, predicting differential autophagy modulation by chloroquine in diabetic subpopulations; meanwhile, magnetically navigated delivery systems elevate myocardial drug uptake to approximately 15% (e.g., miR-24-loaded ferrite nanoparticles traversing microvascular barriers under external magnetic guidance) [132,133]. These translational barriers constitute a tripartite system of hepatic filtration, patient-specific pathophysiological variance, and discordant efficacy assessment, necessitating integrated advances in personalized delivery and clinical endpoint redefinition to bridge the final mile of the translational continuum (Table 3, Ref. [123,124,125,126,127,128,129,130,131]).

**Table 3.** **Clinical translational bottlenecks and conflict**.

Clinical translation bottleneck type	Key evidence	Resolution strategy
Delivery Efficiency [123,124]	Over 90% of nanocarriers are cleared by the liver	Magnetic navigation targeted delivery
Patient Heterogeneity [125,126,127]	The response to calcium overload treatment is reduced by 63% in diabetic patients	iPSC-like organ drug sensitivity testing
Endpoint Deviation [128]	A 68% reduction in infarct area does not improve mortality rate	Composite endpoint: heart failure hospitalization and cardiac resuscitation index
Time Window Delay [129,130,131]	A delay of 3.5 hours in clinical reperfusion leads to the ineffectiveness of NLRP3 inhibitors	Pre-hospital emergency vehicle initiation and microneedle patch preloading

### 4.3 Converging Disciplines and Emerging Horizons

MIRI therapeutics are undergoing transformative cross-disciplinary integration, with three convergent fields spearheading paradigm-shifting advancements. Bioelectronic medicine pioneers novel pathways: implantable vagus nerve stimulators suppress splenic inflammatory cell egress via α7nAChR receptor modulation, reducing myocardial inflammatory cytokines by 52%, while closed-loop sensing platforms enable real-time electrocardiographic monitoring to trigger antiarrhythmic stimulation, effectively constituting an intelligent neuromodulatory platform [134,135,136]. Organ-on-chip and organoid technologies overcome personalized medicine barriers: patient-derived iPSC-based heart-immune dual-cell microphysiological systems successfully model diabetic MIRI pathophysiology, identifying synergistic andrographolide-empagliflozin combinations within microfluidic environments that triple reparative efficacy [137,138,139]. Artificial intelligence accelerates drug repurposing: the deep learning framework AlphaDrug screened 40,000 approved compounds, revealing that the antifungal agent terbinafine attenuates calcium overload through STIM1/Orai1 channel inhibition, with animal studies demonstrating infarct-sparing effects rivaling dedicated calcium blockers [140,141]. Materials-biology fusion yields theranostic implants: conductive hydrogel scaffolds incorporating MXene nanosensors provide mechanical support while continuously monitoring ATP/lactate levels, subsequently releasing SDF-1α via near-infrared responsiveness to recruit stem cells, functioning as bioactive cardiac smart bandages [142,143]. Persistent challenges include infection risks in closed-loop neuromodulatory devices (18% incidence in large-animal models), inadequate organoid vascularization (less than 30% microvascular density), and interspecies efficacy disparities in AI-predicted therapeutics (40% validation rate in humanized models) [144,145]. Breakthroughs in nanorobotics emerge: magnetic iron oxide microparticles self-assemble into microrobotic swarms that traverse microvascular barriers under external magnetic navigation, delivering CRISPR/dCas9 complexes to edit inflammatory genes in ischemic territories, achieving 68% myocardial salvage in rodent models [146,147,148]. This multidisciplinary armamentarium deploys bioelectronic systems as neural conductors, organoid platforms as pathological simulators, AI as computational predictors, and nanorobots as molecular couriers, orchestrating a multidimensional assault on reperfusion injury pathogenesis (Table 4, Ref. [142,144,145,146,147,148]).

**Table 4.** **Contrasting breakthroughs and persistent bottlenecks in converging disciplines**.

Interdisciplinary field	Representative technology	Core breakthroughs	Current limitations
Materials-biology fusion [142]	Closed-loop Neurostimulation and Heart-Immune Dual Cell Microfluidic Chip	Real-time inhibition of inflammation, Anti-arrhythmia and personalized modeling of diabetes mellitus combined with MIRI.	The cost is expensive and the technology has limitations.
AI Drug Rediscovery [144,145]	AlphaDrug Deep Learning Platform	Screening of specific compounds from 40,000 drugs targeting calcium overload	Validation rate of humanized models is only 40%
Nanobots [146,147,148]	Magnetically Controlled Self-assembling Microbots	Targeted delivery of CRISPR to ischemic areas (cardiac salvage rate of 68%)	Challenges in large-scale production

## 5. Limitation

This review has several limitations that warrant acknowledgment. Firstly, this is a narrative review rather than a systematic review. The mechanisms and therapeutic strategies discussed reflect the authors’ scholarly judgment and may not encompass the full breadth of the MIRI literature. Secondly, the manuscript exhibits an inherent imbalance between mechanistic detail and therapeutic application: the molecular pathway sections are extensively developed, while the therapeutic strategies sections remain comparatively limited in scope and depth. This reflects the current state of the field, where mechanistic understanding has advanced more rapidly than clinical translation, but readers seeking comprehensive clinical guidance may find the therapeutic coverage insufficient. The translational relevance of these findings to heterogeneous human patient populations remains uncertain. Finally, this review integrates emerging strategies at the intersection of biological nanotechnology, genome editing, and precision medicine. However, these cutting-edge technologies are rapidly developing, and with the accumulation of new evidence, the therapeutic prospects described in this article may have limitations.

## 6. Conclusion: Advancing Toward Integrative Precision Therapeutics

Research on MIRI has evolved from reductionist examination of isolated pathological mechanisms toward multidimensional network regulation. The discovery of novel programmed cell death modalities including ferroptosis, pyroptosis, and cuproptosis has profoundly broadened conceptual frameworks of cardiomyocyte demise, while spatiotemporal dynamics within the immune microenvironment, emergent mitochondrial signaling paradigms, and intricate non-coding RNA regulatory networks have furnished novel conceptual frameworks for therapeutic design. Future breakthroughs will require the strategic convergence of engineered bionanotechnological delivery systems (e.g., SPIN nanoparticles, stimuli-responsive hydrogels) with multi-omics-guided precision targeting, simultaneously integrating drug repurposing strategies and holistic regulatory paradigms inspired by traditional medicine systems. This integrative approach will ultimately catalyze a paradigm shift from mere vessel recanalization toward comprehensive myocardial preservation. Cross-disciplinary collaboration and innovative technological fusion constitute the imperative pathway for conquering MIRI, a persistent frontier challenge in cardiovascular medicine.
